# Comparing gender-specific suicide mortality rate trends in the United States and Lithuania, 1990–2019: putting one of the “deaths of despair” into perspective

**DOI:** 10.1186/s12888-022-03766-w

**Published:** 2022-02-17

**Authors:** Shannon Lange, Jürgen Rehm, Alexander Tran, Courtney L. Bagge, Domantas Jasilionis, Mark S. Kaplan, Olga Meščeriakova-Veliulienė, Mindaugas Štelemėkas, Charlotte Probst

**Affiliations:** 1 Institute for Mental Health Policy Research, Centre for Addiction and Mental Health (CAMH), 33 Ursula Franklin Street, Toronto, ON M5S 2S1 Canada; 2 Campbell Family Mental Health Research Institute, CAMH, 250 College Street, Toronto, ON M5T 1R8 Canada; 3 Dalla Lana School of Public Health, University of Toronto, 155 College Street, 6th Floor, Toronto, ON M5T 3M7 Canada; 4 Institute of Clinical Psychology and Psychotherapy, Technische Universität Dresden, Chemnitzer Str. 46, 01187 Dresden, Germany; 5 Department of Psychiatry, University of Toronto, 250 College Street, 8th Floor, Toronto, ON M5T 1R8 Canada; 6 Institute of Medical Science, Medical Sciences Building, University of Toronto, King’s College Circle, Room 2374, Toronto, ON M5S 1A8 Canada; 7 Center for Interdisciplinary Addiction Research, Department of Psychiatry and Psychotherapy, University Medical Center Hamburg-Eppendorf, Martinistraße 52, 20246 Hamburg, Germany; 8 Department of International Health Projects, Institute for Leadership and Health Management, I.M. Sechenov First Moscow State Medical University, Trubetskaya str., 8, b. 2, 119992 Moscow, Russian Federation; 9 Department of Psychiatry, University of Michigan Medical School, 2800 Plymouth Road, Ann Arbor, MI 48109 USA; 10 Center for Clinical Management Research, Department of Veterans Affairs, 2215 Fuller Road, Ann Arbor, MI 48105 USA; 11 Laboratory of Demographic Data, Max Planck Institute for Demographic Research, Konrad-Zuse-Str. 1, 18057 Rostock, Germany; 12 Demographic Research Centre, Faculty of Social Sciences, Vytautas Magnus University, Jonavos g. 66, 44191 Kaunas, Lithuania; 13 Luskin School of Public Affairs, University of California, Los Angeles, 337 Charles E Young Drive East, Los Angeles, CA 90095 USA; 14 Department of Health Management, Faculty of Public Health, Lithuanian University of Health Sciences, Tilžės 18, 47181 Kaunas, Lithuania; 15 Health Research Institute, Faculty of Public Health, Lithuanian University of Health Sciences, Tilžės 18, 47181 Kaunas, Lithuania; 16 Department of Preventive Medicine, Faculty of Public Health, Lithuanian University of Health Sciences, Tilžės 18, 47181 Kaunas, Lithuania; 17 Heidelberg Institute of Global Health, Heidelberg University, Im Neuenheimer Feld 130.3, 69120 Heidelberg, Germany

**Keywords:** Gender, Mortality rate, Suicide, Trend

## Abstract

**Introduction:**

The increase in the suicide mortality rate among middle-aged adults in the United States (US) has been well documented. Aside from a few studies from the United Kingdom, it is unclear whether the suicide mortality rate trend in the US is also occurring in other developed countries. Accordingly, we aimed to compare the suicide mortality rate trends over the past 30 years in the US to a country in the European Union–Lithuania.

**Methods:**

Joinpoint regression analyses were performed to identify secular trends in the gender-specific age-standardized suicide mortality rate among individuals 15 + years of age, as well as middle-aged adults (45–54 years of age), and suicide mortality rate ratio for men-to-women.

**Results:**

Age-standardized suicide mortality rates among middle-aged adults in the US increased annually, on average, by 0.89% (95% CI: 0.66%, 1.12%) among men and 1.21% (95% CI: 0.75%, 1.66%) among women between 1990 and 2019. In contrast to the US, there was an overall downward trend in the suicide mortality rates among middle-aged adults in Lithuania across the study period. The average annual percent change in the suicide mortality rate ratio for men-to-women were not statistically significant for either country.

**Conclusion:**

The suicide mortality rate trend in the US does not appear to be an indicator of an upcoming global trend, but rather should be regarded as a cautionary example of what other countries should strive to avoid.

**Supplementary Information:**

The online version contains supplementary material available at 10.1186/s12888-022-03766-w.

## Background

The suicide mortality rate in the United States (US) has been consistently increasing in recent years,[1, 2] with more than 48 thousand people dying by suicide in 2019, for an age-standardized rate of 13.9 per 100,000 people.^2^ Death by suicide has been categorized as one of the “deaths of despair” in the US and is partly responsible for the declining life expectancy.[3] Specifically, Case and Deaton [3, 4] have, famously, shown that the increasing suicide mortality rate, in combination with an increase in other specific causes of death (i.e., accidental poisonings [e.g., opioid overdoses], and alcohol-related liver disease), particularly among middle-aged white adults with a high school degree or less, has been driving life expectancy down. Recently it has been found that the increase in premature death (including death by suicide) is more widespread than originally postulated (e.g., among middle-aged adults of all racial and ethnic groups) [5].

Countries in the UK have also experienced recent rises in middle-aged mortality,[6] for which the increasing number of deaths by suicide among this age group has been identified as a contributory factor.[7] However, it is unclear whether this is also happening in other parts of the world including countries of the European Union (EU). To the best of our knowledge, there has not been a study done to date using advanced statistical methods to compare the suicide mortality rate trend in the US with any country in the EU. Such a comparison could inform us whether or not there are similarities between countries in terms of the cohorts most at risk, which would be suggestive of similar root causes (e.g., underlying social and economic conditions).

As such, it would be interesting to compare the US suicide mortality rate trend over time to that of a country in the EU, such as Lithuania. The focus on the EU was chosen for better comparability, as all EU member states are classified as high income by The World Bank [8]. Yet Lithuania, as the US, is also considered to have high income inequality; [9] considerably higher than in the other EU states with strong social welfare systems such as Nordic countries. While having a gross domestic product purchasing power parity per capita (37,110 international $ in 2020) [8] that is lower than the US (60,138 international $ in 2020) [8], but higher than the majority of countries in Central and Eastern Europe such as Spain and Portugal, Lithuania has one of the highest suicide mortality rates in the world. For instance, in 2016, the age-standardized suicide mortality rate in Lithuania (25.7 per 100,000 people) was more than double the global rate (10.5 per 100,000 people) [10] and over 48% higher than in the EU as a whole. [11] Further, Lithuania had one of the highest suicide mortality rate ratios for men-to-women in the world, at 7.1 in 2016 [12].

Accordingly, the objective of the current study was to compare the suicide mortality rate trends among middle-aged adults (45–54 years of age) specifically, as well as among individuals 15 + years of age overall, over the past 30 years in the US to that in Lithuania, and to identify points of inflection. Identifying points in time when the trends in each country changed will, ideally, prompt hypotheses, as a starting point for further investigation, on the potential factors driving the observed trends. Given the well-recognized universal gender gap in the suicide mortality rate, [13] all analyses were gender-specific.

## Methods

### Data sources

Yearly gender-specific suicide mortality and population data for ages 15 + years by 5-year age groups for the last 30 years (1990 to 2019) were obtained from the Lithuanian Institute of Hygiene and Statistics Lithuania [14] for Lithuania, and the National Vital Statistics System [15] and the US Census Bureau, [16] respectively, for the US.

### Measures

As per the US Centers for Disease Control and Prevention (CDC), [17] suicide is death caused by injuring oneself with the intent to die. As recommended by the CDC when tabulating suicide mortality statistics, the following International Classification of Diseases, 9^th^ revision (ICD-9) and 10^th^ revision (ICD-10), codes were used to ascertain cases of death by suicide in the Lithuanian and US mortality statistics: 950.0–959.9, and X60-X84 and Y87.0, respectively. [18] However, due to coding practices, the ICD-10 code Y87.0 is not used in Lithuania.

### Statistical analysis

For each country and year, the gender-specific number of deaths by suicide by age group was divided by the gender-specific population for each respective age group. The gender- and age-specific yearly death rate, was then multiplied by 100,000 to calculate the suicide mortality rate as deaths per 100,000 people for middle-aged adults (45–54 years of age) specifically, and for individuals 15 + years of age overall. To estimate the standard error (SE) for the gender- and age-specific suicide mortality rate, the following equation was used prior to age-standardization, assuming a Poisson distribution:

$${SE}_{Suicide mortality rate}=\sqrt{\frac{{n}_{gender}}{{{N}_{gender}}^{2}}}$$

where *n* is the gender-specific number of deaths by suicide and *N* is the gender-specific population for each respective year. In order to ensure international comparability, age-standardized suicide mortality rates for individuals 15 + years of age were then computed using the World Health Organization (WHO) standard population.[19] The annual suicide mortality rate ratio for men-to-women was then estimated by dividing the rate among men by the rate among women, and the SE was estimated as follows:

$${SE}_{Rate ratio}=\sqrt{\frac{1}{{n}_{m}}+\frac{1}{{n}_{w}}}$$

In order to identify secular trends in the gender-specific age-standardized suicide mortality rate among middle-aged adults (45–54 years of age) specifically, as well as among individuals 15 + years of age overall and suicide mortality rate ratio for men-to-women, as previously done for elsewhere, [20] joinpoint regression analyses were performed [21]. A joinpoint regression analysis is a data-driven statistical technique that identifies inflection points in the data and 95% confidence intervals (CIs), based on a pre-specified number of joinpoints [21]. For the present analyses a maximum of five joinpoints was specified, as is standard for joinpoint analyses of 30 or more data points [21]. Based on the maximum number of joinpoints, linear segments were fitted to the data. Using a Monte Carlo Permutation method, the fewest number of linear segments such that an additional joinpoint does not add a statistically significant linear trend is selected [21].

Lastly, the slope coefficient for each regression line was transformed to an annual percent change (APC), and the parametric method was used to estimate the 95% CI for the APC of each linear segment. Using a weighted average of the slope coefficients of the underlying joinpoint regression line with the weights equal to the length of each segment over the interval, the average APC (AAPC) over the total study period (1990–2019) was calculated, and the parametric method was used to estimate the respective 95% CI.

Calculations of mortality rates and mortality rate ratios were performed using R version 4.0.2., [22] and the joinpoint regression analyses were conducted using the Joinpoint Regression Program, version 4.8.0.1. [23] Statistical significance was determined using an α of 0.05.

### Results

**Fig. 1:**
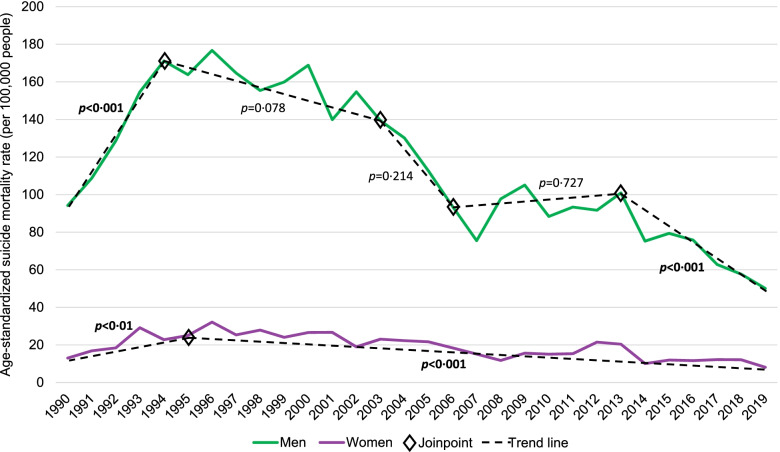
Observed age-standardized^a^ suicide mortality rate (per 100,000 people) and joinpoint trend among middle-aged (45–54 years of age) men and women in the United States, 1990–2019, *Note. P-values are presented for the annual percent change of each linear segment *^a^Standardized to the WHO standard population [19] by 5-year age groups.

**Fig. 2:**
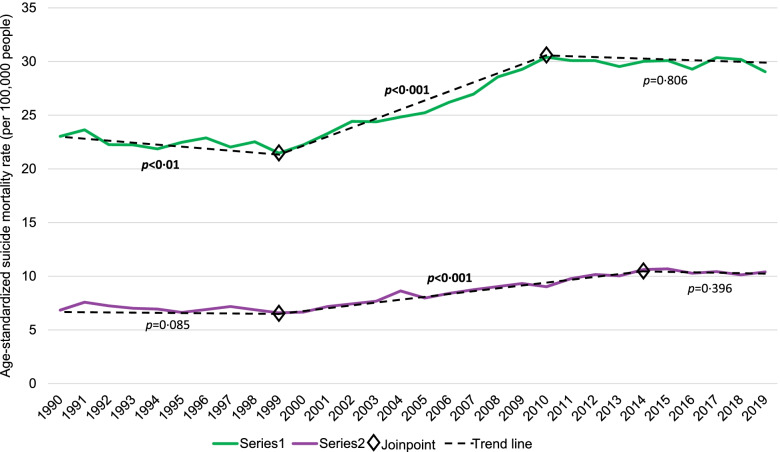
Observed age-standardized^a^ suicide mortality rate (per 100,000 people) and joinpoint trend among middle-aged (45–54 years of age) men and women in Lithuania, 1990–2019, *Note. P-values are presented for the annual percent change of each linear segment.*^a^Standardized to the WHO standard population [19] by 5-year age groups.

Overall, age-standardized suicide mortality rates among middle-aged adults in the US increased annually, on average, by 0.89% (95% CI: 0.66%, 1.12%) among men and 1.21% (95% CI: 0.75%, 1.66%) among women between 1990 and 2019. In contrast to the US, there was an overall downward trend in the suicide mortality rates among middle-aged adults across the total study period in Lithuania, albeit the AAPC was not statistically significant (Table 1). The suicide mortality rate among middle-aged adults in the US reached its highest point in the mid-to-late 2000s – in 2017 for men at 30.4 per 100,000 men and 2015 for women at 10.7 per 100,000 women. Compared to the US, at its highest point (in 1996), the suicide mortality rate among middle-aged adults in Lithuania was nearly six times higher for men and three times higher for women – at 176.7 per 100,000 men and 32.1 per 100,000 women. However, when the US was experiencing its highest suicide mortality rate in the last 30 years, Lithuania was, for the most part, experiencing its lowest rate. See Figs. 1 and 2 for the gender-specific age-standardized suicide mortality rates among middle-aged adults in the US and Lithuania, respectively, by year for the past 30 years.

**Table 1:** Estimates from joinpoint analyses of age-standardized^a^ suicide mortality rates (per 100,000 people) for middle-aged (45–54 years of age) men and women in the United States and Lithuania, 1990–2019

	Mortality rate per 100,000 people		Total study period^b^			Period 1			Period 2			Period 3			Period 4			Period 5	
	1990	2019	AAPC (%)	95% CI	Years	APC (%)	95% CI	Years	APC (%)	95% CI	Years	APC (%)	95% CI	Years	APC (%)	95% CI	Years	APC (%)	95% CI
United States																			
Men	23.03	29.05	0.89^d^	0.66, 1.12	1990–1999	-0.61^d^	-1.05, -0.17	1999–2010	2.92^d^	2.54, 3.30	2010–2019	-0.05	-0.49, 0.39	-	-	-	-	-	-
Women	6.84	10.40	1.21^d^	0.75, 1.66	1990–1999	-0.68	-1.46, 0.10	1999–2014	3.06^d^	2.65, 3.46	2014–2019	-0.85	-2.84, 1.19	-	-	-	-	-	-
Men-to-women^c^	3.37	2.79	-0.29	-1.53, 0.97	1990–2001	0.39	-0.13, 0.91	2001–2004	-2.89	-12.52, 7.80	2004–2010	1.15	-0.68, 3.02	2010–2014	-3.42	-7.66, 1.02	2014–2019	0.63	-1.21, 2.51
Lithuania																			
Men	94.19	50.07	-2.04	-4.84, 0.84	1990–1994	16.59^d^	8.97, 24.74	1994–2003	-1.86	-3.91, 0.23	2003–2006	-14.83	-34.52, 10.78	2006–2013	0.56	-2.75, 4.00	2013–2019	-9.52^d^	-12.54, -6.40
Women	12.99	8.11	-1.01	-2.94, 0.96	1990–1995	16.19^d^	3.93, 29.90	1995–2019	-4.26^d^	-5.15, -3.36	-	-	-	-	-	-	-	-	-
Men-to-women^c^	7.25	6.17	-0.52	-1.04, 0.01	1990–2019	-	-	-	-	-	-	-	-	-	-	-	-	-	-

*APC*, Average annual percent change, *APC*, Annual percent change *CI*, Confidence interval, ^a^Standardized to the WHO standard population [19] by 5-year age groups, ^b^Years 1990–2019, ^c^Rate-ratio of the age-standardized suicide mortality rate, ^d^Statistically significant (*p* < 0.05)

Joinpoint regression analyses resulted in two points of inflection for the age-standardized suicide mortality rate among both middle-aged men and women in the US, with a significant decrease in the first period followed by a significant increase in the second period for men and a significant increase in the second period for women (Table 1 and Fig. 1). Specifically, following a significant decrease from 1990 to1999 (APC = -0.60%; 95% CI: -1.05%, -0.17%), the suicide mortality rate among middle-aged men in the US significantly increased from 1999 to 2010 (APC = 2.92%; 95% CI: 2.54%, 3.30%) and thereafter remained relatively unchanged. The suicide mortality rate trend among middle-aged women was similar to that among men. The rate increased significantly from 1999 to 2014 (APC = 3.06%; 95% CI: 2.65%, 3.46%), following a period of relatively little change, and thereafter stabilized.

**Fig. 3:**
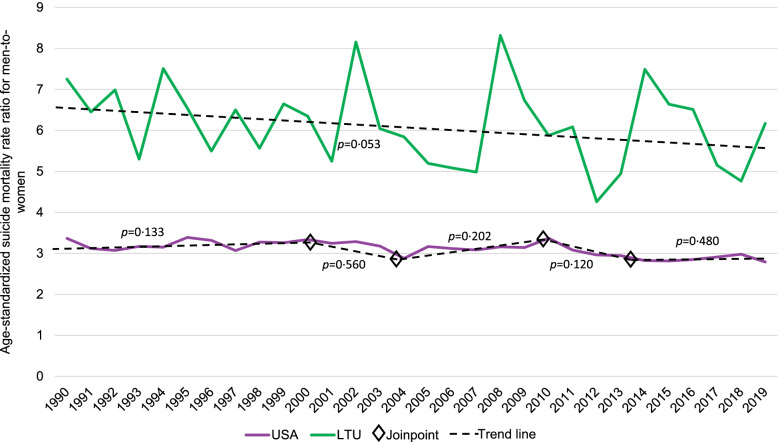
Observed age-standardized^a^ suicide mortality rate ratio for men-to-women and joinpoint trend among middle-aged adults (45–54 years of age) in the United States and Lithuania, 1990–2019, *Note. P-values are presented for the annual percent change of each linear segment*

Among middle-aged adults in Lithuania, joinpoint regression analyses resulted in four inflection points for the age-standardized suicide mortality rate among men and one point of inflection among women (Table 1 and Fig. 2). Among middle-aged men in Lithuania, there was a significant increase (APC = 16.59%; 95% CI: 8.97%, 24.74%) in the first period (1990 to 1994) and a significant decrease (APC = -9.52%; 95% CI: -12.54%, -6.40%) in the fifth period (2013 to 2019); between the first and fifth periods, there was relatively long period of instability (as indicated by three periods with non-significant APCs, varying in direction). The suicide mortality rate trend among middle-aged women in Lithuania was much less dynamic, with a significant increase (APC = 16.19%; 95% CI: 3.93%, 29.90%) in the first period (1990 to 1995) followed by a significant decrease (APC = -4.26%; 95% CI: -5.15%, -3.36%) in the second period (1995 to 2019). Despite large fluctuations in the suicide mortality rate ratio for men-to-women in Lithuania, which ranged from 4.85 (in 1992) to 7.44 (2016), no inflection points were identified and the average annual percent decrease was not statistically significant (Fig. 3 and Table 1). Compared to Lithuania, the US had a lower suicide mortality rate ratio for men-to-women consistently over the past 30 years (ranging from 3.43 [in 2015] to 4.64 [in 1995]). Despite the joinpoint analysis resulting in four inflection points for the rate ratio in the US, none of the linear segments had a statistically significant APC, nor was the AAPC significant (-0.29%; 95% CI: -1.53%, 0.97%).

The suicide mortality rate trends among individuals 15 + years of age are presented in the Appendix. Despite middle-aged adults having higher suicide mortality rates than individuals 15 + years of age in both the US and Lithuania, the suicide mortality rate trends among individuals 15 + years of age are comparable to the trends among middle-aged adults.

## Discussion

The current findings suggest that the suicide mortality rate trend in the US is not necessarily an indicator of an upcoming global trend and may in fact be atypical, as Case and Deaton [3] and others [24] have postulated with respect to the so-called deaths of despair in general. Even though a similar trend could be occurring elsewhere (for example, see [7]), it appears to be restricted to certain countries. Generally speaking, we found that in the US, gender-specific suicide mortality rates are increasing, while in Lithuania the rates are decreasing. The suicide mortality rate in the US has received a lot of attention in recent years, with much of the focus being on middle-aged adults since the publication of Case and Deaton’s work [3, 4]. However, when compared to a country such as Lithuania, where despite substantial progress since the mid-2000s, the suicide mortality rates remain among the highest in the world, the rates do not seem as alarming as they do in the US-centric literature. Yet the increasing suicide mortality rate trend among men and women in the US, in contrast to systematic reduction in Lithuania, should be a matter of concern.

The finding of country-specific suicide mortality trends and inflection points suggests that there have been country-specific shifts in cultural, political, social and/or economic factors and/or specific individual-level risk factors that are acting differentially on certain populations, whether it is a change in their prevalence or in the risk relationship over time. As indicated, the overall focus of the current study was to determine whether the US is a bellwether with respect to death by suicide. The next step, however, is a difficult endeavor – determining what factors are influencing the observed trends and how to counter them, which is where the inflection points identified should prove to be useful. Accordingly, the current study is intended to evoke hypotheses and spark further investigation into socio-cultural factors and their impact on suicide mortality risk, as well as gender-specific risk relationships for suicide mortality and how they have changed over time.

Death by suicide is a complex phenomenon like all mental health outcomes, arising from many factors (e.g., demographic, economic, neighbourhood, environmental events, and social and cultural), both on the proximal and distal levels. [25] A review of potential factors that could impact suicide and strategies/interventions to prevent suicide mortality is beyond the scope of this paper (for a review see [26]). However, we will describe one example, problematic substance use, to illustrate how a risk factor and public policy could influence temporal trends. Problematic substance use has been identified as an important behavioural risk factor for death by suicide that is strongly influenced by social and cultural factors. At the macro-level effective public health policies targeting substance use could reduce the suicide mortality rate in the respective country [27] For example, in Lithuania several alcohol control policies have been implemented since 2008, one of which was a major increase in taxation in 2017. This increase in taxation, which lead to a decrease in alcohol affordability, was found to have significantly reduced the suicide mortality rate among men (the suicide mortality rate for women was not significantly impacted). [28] In comparison, although alcohol control policies have been implemented in the US within the past 30-years at the state level, the lack of national-level implementation [29] could partly account for the increasing suicide mortality rate.

To our knowledge, this is the first study to use joinpoint regression analyses to identify specific points of inflection and the linear trend in-between for the gender-specific suicide mortality rate for the past 30 years in the US and to draw comparisons with a high-income European country. Among the strengths of the current study is the long time period (1990–2019) covered. Further, the analytical method of choice – joinpoint regression analysis – allowed us to test whether a multi-segmented line best fit the data, as compared to a straight line, which provides a much more detailed overview of what has been happening with respect to suicide mortality rates over time than a single summary trend statistic, for example. However, there are a few limitations that should be acknowledged. First, joinpoint regression analysis fits linear segments to data, which may or may not be linear. Even though the identified points of inflection provide a useful approximation of the year in which the gender-specific suicide mortality rate trend changed significantly, they represent a simplification of the observed secular trend. They should therefore be interpreted with a certain degree of uncertainty. Second, while postulated that trends in “deaths of despair” vary by level of education, [4] it was not possible to pull in such individual-level data in the current investigation. Third, it should be acknowledged that there is the potential of misclassifying suicides as drug overdoses, which would result in an underestimate of the suicide mortality rate (for example, see [30]). The number of misclassified cases may be heightened during the current opioid epidemic in the US, which overlaps with the current study period. Lastly, although it has been reported elsewhere that gender-specific suicide mortality rates are on the rise among middle-aged adults in the US, we found that since 2010 for men and 2014 for women, the rates have remained relatively stagnant. This could be due to using the WHO standard population to produce age-standardized estimates, a prerequisite for international comparisons, rather than the US population.

## Conclusion

Prevention of death by suicide has been recognized at both the national [31] and international [32, 33] level; however, little progress has been made in the US. Considering the trend differences in suicide mortality rates between men and women, highlighted by the current study, prevention and intervention strategies should be tailored to target gender-specific aspects. Such strategies should be culturally relevant, as it is evident that gender-specific suicide mortality trends can drastically differ from one country to the next.

## Patient and public involvement

Patients or the public were not involved in the design, or conduct, or reporting, or dissemination plans of our research.

## Supplementary Information

**Additional file 1.** Supplementary Material.

## Data Availability

The original data are administrative data of the US and Lithuanian government agencies, and can be obtained directly from the original sources (exact sources are indicated in the article). The R program code for all analyses carried out will be made available via github.com, following publication.

## Abbreviations

US: United States
APC: Annual percent change
AAPC: Average annual percent change
SE: Standard error
CI: Confidence interval
WHO: World Health Organization
