# Supplementary material for: Comparing gender-specific suicide mortality rate trends in the United States and Lithuania, 1990–2019: putting one of the “deaths of despair” into perspective
Source: BMC Psychiatry. 2022 Feb 17;22:127. doi: 10.1186/s12888-022-03766-w (PMC8851770; doi:10.1186/s12888-022-03766-w)
Supplement: Supplementary file 1 — Additional file 1. Supplementary Material. [file 12888_2022_3766_MOESM1_ESM.docx]

**Supplementary Material**

**Table A1.** Estimates from joinpoint analyses of age-standardized^a^ suicide mortality rates (per 100,000 people) among men and women 15+ years of age in the United States and Lithuania, 1990-2019

|  | **Mortality rate per 100,000 people** | | **Total study period^b^** | | **Period 1** | | | **Period 2** | | | **Period 3** | | | **Period 4** | | | **Period 5** | | |
| --- | --- | --- | --- | --- | --- | --- | --- | --- | --- | --- | --- | --- | --- | --- | --- | --- | --- | --- | --- |
|  | **1990** | **2019** | **AAPC (%)** | **95% CI** | **Years** | **APC (%)** | **95% CI** | **Years** | **APC (%)** | **95% CI** | **Years** | **APC (%)** | **95% CI** | **Years** | **APC (%)** | **95% CI** | **Years** | **APC (%)** | **95% CI** |
| United States | | | | | | | | | | | | | | | | | | | |
| Men | 25·60 | 27·43 | 0·31 | -0·05, 0·68 | 1990-1995 | -0·71 | -1·67, 0·27 | 1995-1999 | -3·23^d^ | -5·20, -1·21 | 1999-2006 | 0·36 | -0·38, 1·11 | 2006-2019 | 1·80^d^ | 1·56, 2·05 | - | - | - |
| Women | 5·93 | 7·39 | 0·85^d^ | 0·56, 1·16 | 1990-2000 | -1·84^d^ | -2·12, -1·56 | 2000-2009 | 2·27^d^ | 1·86, 2·68 | 2009-2016 | 3·52^d^ | 2·86, 4·18 | 2016-2019 | -0·38 | -2·55, 1·83 | - | - | - |
| Men-to-women^c^ | 4·32 | 3·71 | -0·51^d^ | -0·86, -0·15 | 1990-1995 | 1·37^d^ | 0·09, 2·68 | 1995, 2016 | -1·30^d^ | -1·47, -1·14 | 2016-2019 | 1·99 | -0·75, 4·80 | - | - | - | - | - | - |
| Lithuania | | | | | | | | | | | | | | | | | | | |
| Men | 52·22 | 40·46 | -1·08 | -2·84, 0·71 | 1900-1994 | 16·55^d^ | 11·83, 21·47 | 1994-2003 | -1·32^d^ | -2·58, -0·04 | 2003-2006 | -9·43 | -23·07, 6·63 | 2006-2014 | -0·77 | -2·33, 0·82 | 2014-2019 | -8·59^d^ | -11·12, -5·98 |
| Women | 9·93 | 6·35 | -1·77 | -4·90, 1·46 | 1990-1995 | 8·88^d^ | 3·85, 14·16 | 1995-2004 | -2·61^d^ | -4·64, -0·54 | 2004-2007 | -9·78 | -33·26, 21·97 | 2007-2013 | 1·26 | -3·29, 6·02 | 2013-2019 | -7·56 | -10·70, -4·30 |
| Men-to-women^c^ | 5·26 | 6·37 | 0·32 | -0·04, 0·67 | - | - | - | - | - | - | - | - | - | - | - | - | - | - | - |

APC: Average annual percent change; APC: Annual percent change; CI: Confidence interval

^a^Standardized to the WHO standard population by 5-year age groups

^b^Years 1990 to 2019

^c^Rate-ratio of the age-standardized suicide mortality rate

^d^Statistically significant (*p*<0·05)

**Figure A1.** Observed age-standardized^a^ suicide mortality rate (per 100,000 people) and joinpoint trend among men and women 15+ years of age in the United States, 1990-2019

*Note.* *P-values are presented for the annual percent change of each linear segment.*

^a^Standardized to the WHO standard population by 5-year age groups.

**Figure A2.** Observed age-standardized^a^ suicide mortality rate (per 100,000 people) and joinpoint trend among men and women 15+ years of age in Lithuania, 1990-2019.

*Note.* *P-values are presented for the annual percent change of each linear segment.*

^a^Standardized to the WHO standard population by 5-year age groups.

**Figure A3.** Observed age-standardized^a^ suicide mortality rate ratio for men-to-women and joinpoint trend among individuals 15+ years of age in the United States and Lithuania, 1990-2019.

*Note.* *P-values are presented for the annual percent change of each linear segment.*

^a^Standardized to the WHO standard population by 5-year age groups.
